# Gaussian accelerated molecular dynamics simulations facilitate prediction of the permeability of cyclic peptides

**DOI:** 10.1371/journal.pone.0300688

**Published:** 2024-04-23

**Authors:** Nicolas Frazee, Kyle R. Billlings, Blake Mertz

**Affiliations:** C. Eugene Bennett Department of Chemistry, West Virginia University, Morgantown, WV, United States of America; International Centre for Genetic Engineering and Biotechnology, INDIA

## Abstract

Despite their widespread use as therapeutics, clinical development of small molecule drugs remains challenging. Among the many parameters that undergo optimization during the drug development process, increasing passive cell permeability (i.e., log(P)) can have some of the largest impact on potency. Cyclic peptides (CPs) have emerged as a viable alternative to small molecules, as they retain many of the advantages of small molecules (oral availability, target specificity) while being highly effective at traversing the plasma membrane. However, the relationship between the dominant conformations that typify CPs in an aqueous versus a membrane environment and cell permeability remain poorly characterized. In this study, we have used Gaussian accelerated molecular dynamics (GaMD) simulations to characterize the effect of solvent on the free energy landscape of lariat peptides, a subset of CPs that have recently shown potential for drug development (Kelly *et al.*, *JACS* 2021). Differences in the free energy of lariat peptides as a function of solvent can be used to predict permeability of these molecules, and our results show that permeability is most greatly influenced by N-methylation and exposure to solvent. Our approach lays the groundwork for using GaMD as a way to virtually screen large libraries of CPs and drive forward development of CP-based therapeutics.

## Introduction

Drug development remains a challenge for the vast majority of treatments due to a variety of factors, including oral availability, optimization for specificity to the intended target, and minimization of off-target effects and toxicity [1, 2]. Small molecules are often the first choice in drug development because of oral administration, but they often have significant side effects (e.g., lack of permeability [3], high clearance [4], toxicity [5]), which have indirectly led to increasing adoption of alternative drug vehicles such as monoclonal antibodies [6], chimeric antigen receptor T-cells [7], and ubiquitin ligase targeted degraders [8], to name a few. Cyclic peptides (CPs) lie between the chemical space of small molecules and antibody-based therapeutics but possess the advantages of both classes: they can be orally administered, are non-toxic, membrane permeable, and have the ability to target protein-protein interactions [9–12]. However, CPs also have their own set of drawbacks. They often require lengthy synthetic pathways, making it much more difficult to conduct large-scale screens in comparison to small molecule libraries that are often on the scale of millions to billions of compounds. In addition, prediction of ADME (absorption, distribution, metabolism, and excretion) properties remains challenging, as CPs don’t follow Lipinsky’s rule of 5 [13, 14]. Despite these challenges, a number of naturally-occurring and synthetic CPs have been adapted for clinical use (e.g., vancomycin [15], cyclosporin A [16], melanotan II [17]).

Optimization of CPs typically focuses on permeability and prevention of degradation. N-methylation is a common practice to improve CP permeability [18]. Incorporation of D-amino acids has been shown to enhance resistance to proteolytic degradation [19] by inducing compact conformations, a characteristic that is directly correlated with cell permeability [20, 21]. Until very recently, lariat peptides (i.e., cyclic peptides with tail-to-sidechain cyclization, termed a depsipeptide linkage) have received little attention for therapeutic development. Lariat peptides comprise almost a third of naturally-occurring CPs, making them a potentially promising vehicle for drug development. Unlike conventional CPs, the tail on a lariat peptide imbues them with unique characteristics such as the ability to tightly bind to extended protein-protein interactions (PPIs) [9, 22].

One of the underlying hypotheses for the effective cell permeability of CPs is that their unique backbone configuration allows the peptide to adopt polar or non-polar conformations, thus allowing the molecule to convert to the conformation that corresponds to the surrounding environment and facilitating passive diffusion [11, 21, 23]. Traditionally, the effectiveness of diffusion of CPs has been measured via water-to-octanol partition coefficients (log*P*), but with the advent of large CP libraries, it has become more advantageous to use the parallel artificial membrane permeability assay (PAMPA), which allows for rapid quantification of membrane permeability that is on par with partitioning coefficients [24].

Modeling of cyclic peptides and their accompanying permeability has long been a challenging task for the computational community. One of the most effective theoretical approaches uses a size-based reweighting to generate estimates of log*P* [25]. Machine learning (ML) approaches have rapidly improved in their ability to predict cyclic peptide structures [26, 27] but still require structural data for validation. Free energy molecular dynamics (MD) simulations provide the most detailed physical description of diffusion of CPs across a model bilayer [11, 28] but are computationally expensive. An inherent need exists for a physics-based computational tool that is cost-effective. Inspired by past studies that have used organic solvents as membrane mimetics [29] and the ability of accelerated molecular dynamics to efficiently sample the conformational space of CPs [30], we propose to combine these two approaches to rapidly and effectively predict cell permeability of lariat peptides.

## Materials and methods

### Force field parameterization

The CHARMM36 force field was used for all molecules unless otherwise specified [31]. The TIP3P model [32] was used for aqueous solvent and the CGENFF force field was used for octanol solvent [33]. Topologies for end-to-end cyclic peptide linkages existed in the CHARMM36 force field (unpublished) and were adapted for use in depsipeptide linkages. Force field parameters for the depsilinkage (ester linkage) between Thr3 and Pro9 were generated via the FFTK plugin in VMD [34]. Parameters for methylated leucine, methylated D-leucine, and methylated D-alanine were determined by comparison to existing residues in the CHARMM36 protein force field [35].

### Structure generation

**Fig 1:**
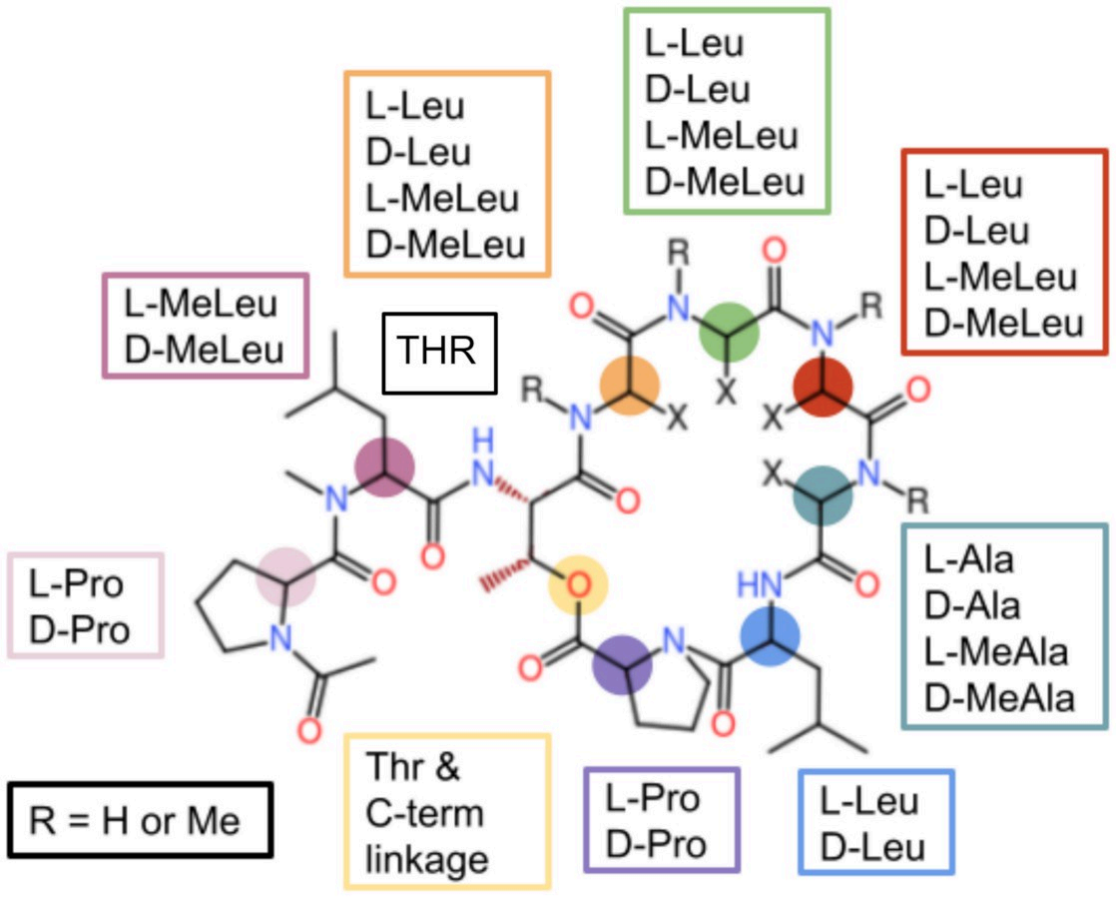
Cyclic peptide scaffold used in this study. Lariat peptides have a short tail in addition to a cyclic backbone. In this particular study, peptides had seven residues within the macrocycle connected by a depsi linkage between the sidechain of Thr3 and the C-terminus of Pro9. The peptide library consists of permutations at each residue: “L” and “D” designate the specific amino acid enantiomers and “Me” designates methylation of the backbone nitrogen.

Fifteen peptides were chosen randomly from each of the sixteen sub-libraries tested in Kelly et al. [22], resulting in 240 total peptides (Fig 1). Of the 240 peptides that were generated, only 89 had corresponding experimental data, due to the fact that the synthesis protocol employed by Lokey and coworkers produced fewer lariat peptides than the theoretical maximum of 4096 [22]. Linear structures were generated and the depsipeptide linkage was formed with psfgen in VMD [36]. Peptide structures were minimized in NAMD 2.14 [37] for 5000 steps with a 10 kcal/mol/Å^2^ restraint on the omega dihedrals of the residues in the ring using colvars [38]. Each peptide was then solvated in water or in octanol using the *solvate* plugin in VMD [36].

### Molecular dynamics simulations

Solvated systems were minimized for 7500 steps and then equilibrated in the *NPT* ensemble (*P* = 1 atm, *T* = 310 K) for 100 ps, with production runs in the *NVT* ensemble (*T* = 310 K) for 10 ns of conventional MD (cMD) followed by 40 ns using the Gaussian accelerated MD (GaMD) [39–41] implementation in NAMD 2.14 [42]. GaMD is an enhanced sampling method that accelerates conformational transitions of biomolecules by adding a boost potential to the energy in a molecular mechanics force field

$$\begin{array}{c} \Delta V\left(r\right)=\frac{1}{2}k{\left(E-V\left(\vec{r}\right)\right)}^{2} \end{array}$$ (1)

whenever the system potential $V\left(\vec{r}\right)$ is less than a user-specified threshold energy *E* (and also scaled by the harmonic force constant *k*). The distribution of the standard deviation of Δ*V* is kept small enough to ensure accurate reweighting of the potential energy surface using cumulant expansion. A boost potential term can be added to the overall potential energy as well as the dihedral potential energy (i.e., “dual-boost” potential) in order to maximize sampling of the conformational landscape. We used dual-boost GaMD for all simulations in this study. Further details on the theory behind GaMD can be found in Maio, Feher, and McCammon [39].

A total of 480 simulations were completed (240 peptides in the two solvent systems of water or octanol). To validate that our systems had converged within 50 ns of combined cMD and GaMD, four cyclic peptide systems were run under the same conditions for 250 ns. No changes in peptide conformation was observed after 50 ns, leading to our final production simulation time.

### Calculation of peptide permeability

The Stokes-Einstein equation was used to calculate the diffusion coefficient for each peptide:

$$\begin{array}{c} D=K_{B}T/6\pi \eta R_{o} \end{array}$$ (2)

where *D* relates the thermal energy (the product of the Boltzmann constant *K*_*B*_ and the average temperature of the simulation *T* in Kelvin) to the solute radius *R*_*o*_ and viscosity *η*. The diffusion coefficient from Eq (1) can then be used to derive the permeability of a given cyclic peptide:

$$\begin{array}{c} \frac{1}{P}\;=R=\int_{z_{aq}}^{z_{oct}}\;\text{exp}\;\frac{\beta W(z)}{D(z)}\;dz \end{array}$$ (3)

where the resistivity, *R*, is the inverse of the the permeability *P*. The upper and lower boundary conditions of the integral represent the conformation of the peptide in a given solvent. The exponential relationship is the quotient of the inverse of the Boltzmann distribution (1/*K*_*B*_*T*) and the PMF of the peptide *W*(*z*) with the diffusion coefficient *D*. The permeability relationship 2 was adapted from [43].

The rmsd2ref and PyLOOS tools from LOOS [44] were used to carry out the calculations to obtain *W*(*z*) and *R*_*o*_, respectively. Results from rmsd2ref were grouped using K-means clustering, after which the respective populations in each cluster were used to assign weights for calculation of the PMF. PyLOOS was used to find the smallest sphere that can fit the average conformation of the protein to find the radius *R*_*o*_.

### Analysis

The backbone dihedral angles were measured using the torsion tool in LOOS [44]. The depsipeptide linkage interrupts the measurement of the final *ω* dihedral. Solvent accessible surface areas (SASA) of the backbone oxygens and of the entire peptide were measured in VMD [36] using a probe radius of 1.4 Å. *Hbonds.* The number of hydrogen bonds was measured using the hbonds tool in LOOS [44]. Principle component analysis(PCA) of the heavy atoms of each respective peptide was performed using LOOS. All plots were produced with either gnuplot, matplotlib, or VMD.

## Results

**Fig 2:**
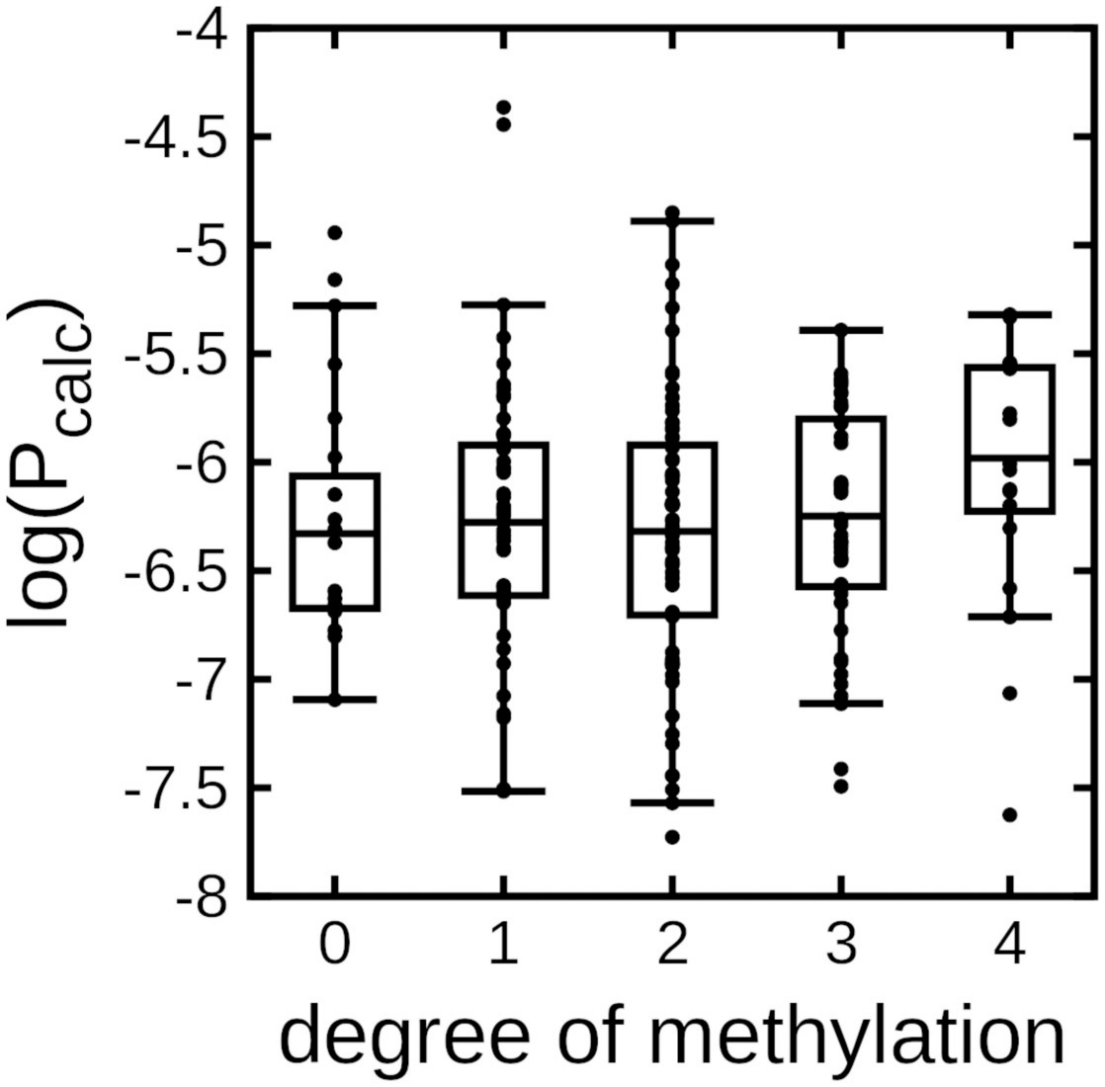
N-methylation generally correlates with permeability. Box plot of partition coefficients based on MD simulations (*P_calc_*) as a function of the number of N-methylations on the cyclic peptide backbone. Mid-points in each box is the mean for each particular data set. Boxes represent the two intermediate quartiles of each data set. Boxes and means were calculated by leaving out the two largest outliers.

Although the range of log(P_calc_) is similar to that of log(P_app_), no general correlation exists for the entire set of peptides that were modeled (S1 Fig). (The Pearson correlation coefficient was -0.0059; correlated or anti-correlated data sets have values that exceed +/- 0.5). This initially indicates that calculation of partitioning coefficients based on the PMF between two solvents alone is insufficient to correctly predict behavior of permeability of cyclic peptides. The next step was to identify other observables from our simulations that could indicate differences in behavior of cyclic peptides as a function of solvent. We observe fairly good agreement with the experimental results of Kelly et al. [22] as a function of the number of N-methylations, in which there is a noticeable increase in permeability from *N* = 0 to *N* = 1 and a slight increase from *N* = 1 to *N* = 4 (Fig 2). For each N atom that is methylated, it removes a potential hydrogen bond donor from the molecule. The conventional hypothesis is that addition of N-methyl groups effectively makes the cyclic peptide less soluble in aqueous solution and more prone to diffuse across the membrane.

In order to more appropriately represent the distribution of log(P) values for each methylation state, we dropped the two farthest outliers from each set of data points. Careful analysis of the lariat peptide library from [22] shows two things: 1) N-methylation is a gaussian distribution (most highly populated value is *N* = 2), and 2) actual experimental data on the peptide library is 23–40% of the total theoretical number (S2 Fig). *N* = 0 and *N* = 4 were by far the most poorly represented peptides [22], and so it was necessary for them to over-sample these two populations in order to capture accurate permeability behavior. In our case, we did not have the computational resources to run an equivalent number of cyclic peptides (this would have translated to an additional 30–40 peptide systems), and so the permeability behavior for *N* = 0, 4 will be noisier than for *N* = 1, 2, and 3. By removing the two largest outliers, we more effectively capture the trends across the entire library tested, leading to greater agreement with the experimental data.

Comparison to [22] shows that permeability is loosely correlated with heterochirality (S3 Fig). However, this may have been an optimistic interpretation of their results in light of our data. For the more largely populated portions of heterochirality (*N* = 2,3,4,5, S4 Fig), the distributions were quite broad (log(P_app_) -5.0 to -7.5); our data is roughly consistent with these results. (Comparison between experimental and computational results for *N* = 0, 1, 7, and 8 would largely be speculative because of the smaller number of samples for each of these states). Given these conditions, the most likely conclusion to draw from our results is that there is little to no correlation between heterochirality and permeability. This underscores the need to employ additional approaches to effectively identify promising candidates.

**Fig 3:**
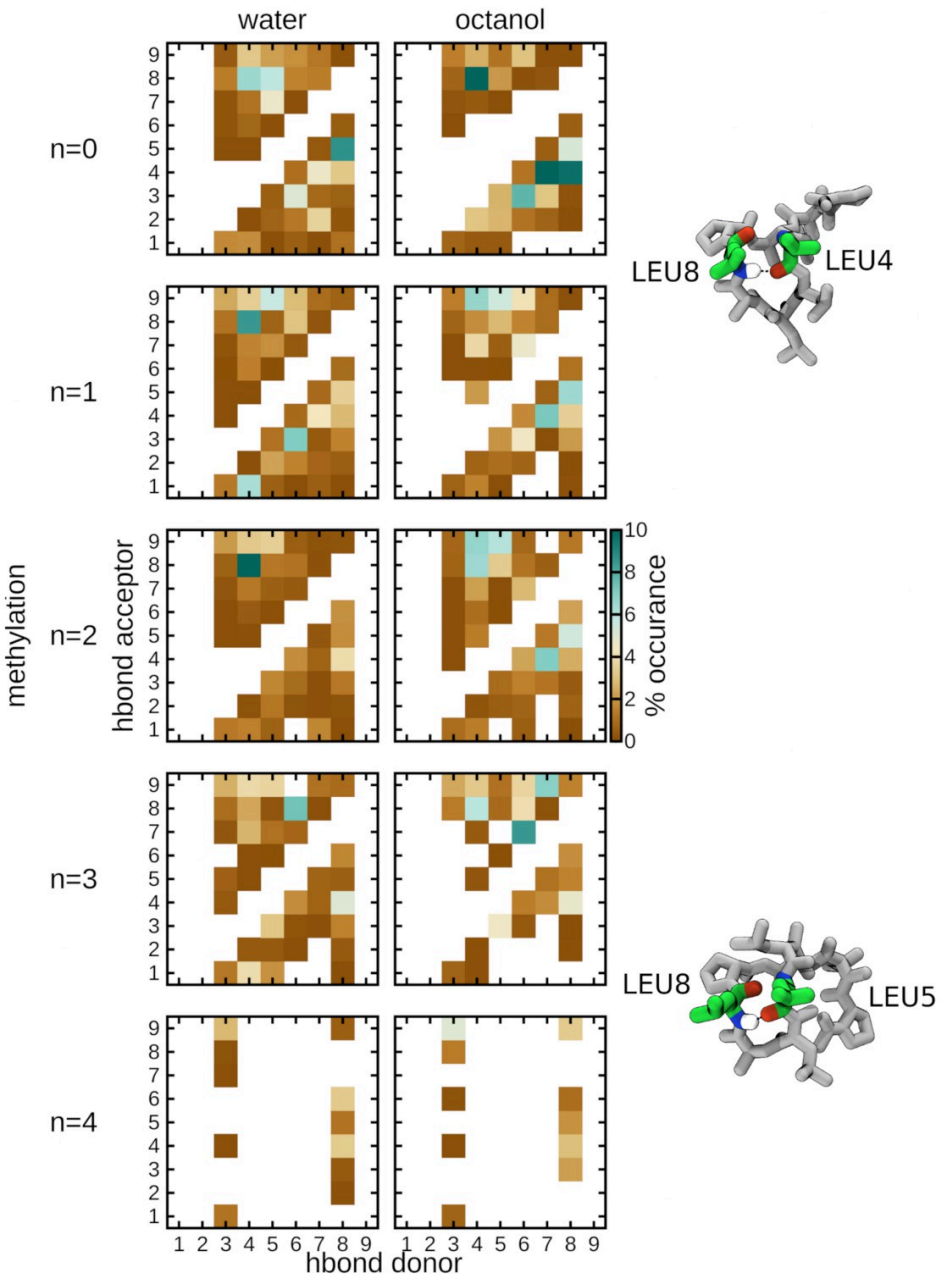
Octanol solvent is a major driver of formation of intramolecular hydrogen bonds. Fraction of intramolecular hydrogen bond formation between the donor (NH) and acceptor (C = O) groups in cyclic peptides as a function of N-methylation (*N* = 0, 1, 2, 3, 4) and solvent (water or octanol). White indicates the complete absence of interaction.

In general, the propensity for hydrogen bond formation decreases with an increase in degree of N-methylation (Fig 3). In particular, Leu8 consistently participates as a hydrogen bond acceptor regardless of solvent, indicating its importance as a key residue towards stabilization of the cyclic peptides. Quite often, Leu4 and Leu5 are the hydrogen bond donors to Leu8 which can stabilize particular conformations of the cyclic peptide backbone. A noticeable difference between solvents occurs in octanol, where Leu4 and Leu5 participate as hydrogen bond acceptors as well, forming hydrogen bonds with Ala7 and Leu8. This additional non-bonded interaction may be necessary to facilitate the conformational transition that allows for effective diffusion of these specific cyclic peptides from a polar to a nonpolar environment.

**Fig 4:**
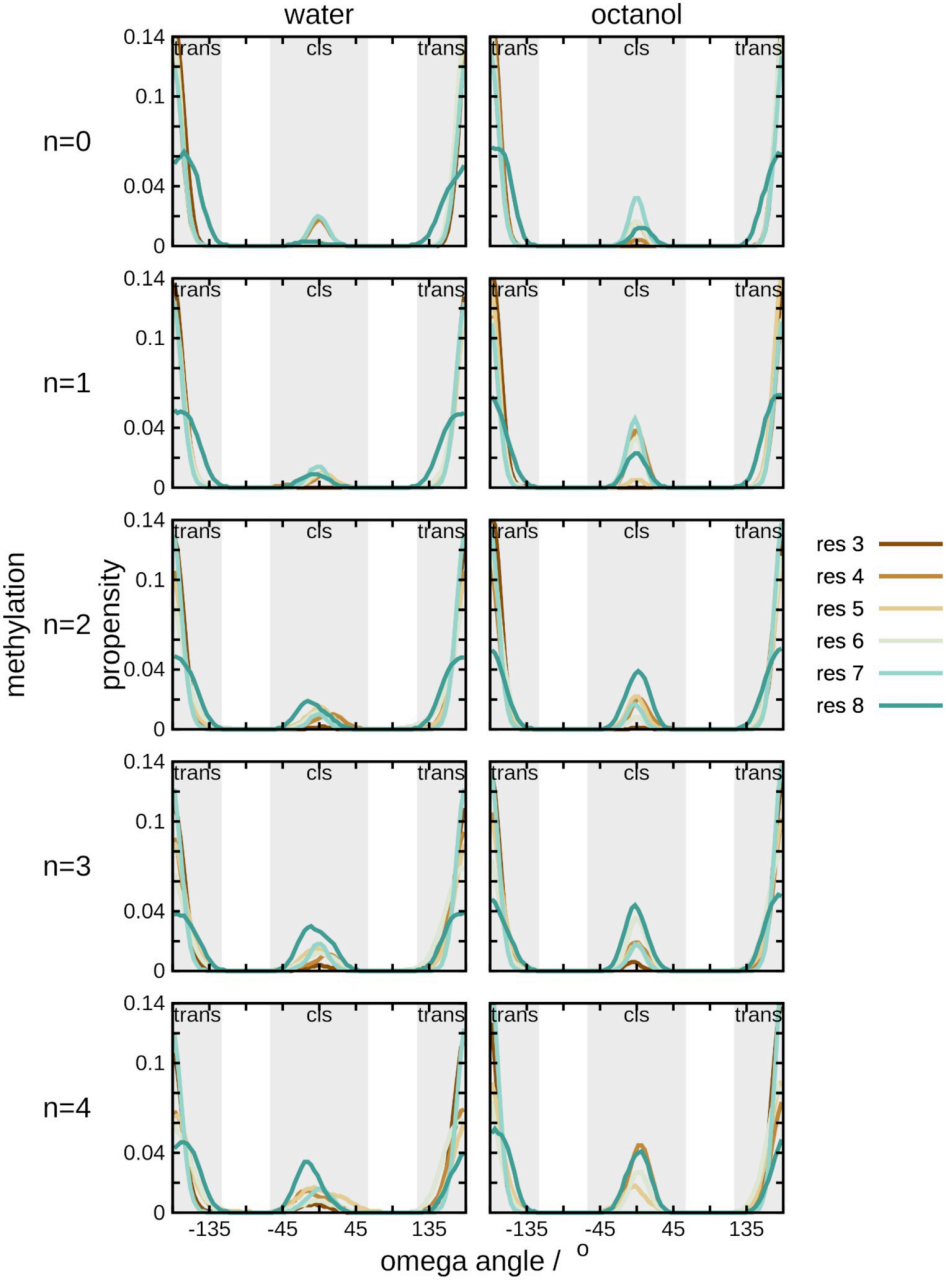
Solvent type and degree of methylation shifts the conformational equilibrium of lariat peptides. Per-residue normalized distribution of omega dihedral angles of the lariat peptides tested here. *First column*: water; *second column*: octanol; *rows*: increasing degrees of N-methylation.

Two trends can be observed here. The first is solvent-dependent; for cyclic peptides with lower degrees of methylation (*N* = 0, 1, and 2), solvation in octanol leads to a higher propensity to sample *cis* conformations within the backbone (Fig 4). This is not necessarily consistent by residue position; however, residues 7 (Leu) and 8 (Pro) tend to have a higher fraction of sampling in the *cis* conformation than other residues in the peptides. The second trend correlates with the degree of N-methylation. As methylation of the lariat peptides increases, sampling of *cis* backbone conformations increases. This effect is more noticeable than the solvent effect, as can be seen for *N* = 3, 4; the probability density for sampling of *cis* conformations is nearly equivalent between aqueous and organic solvents. Essentially, the contributions of solvent and degree of methylation to conformational sampling is a balance between enthalpy and entropy. As methylation increases the cyclic peptide backbone is more sterically restricted (i.e., entropy decreases).

**Fig 5:**
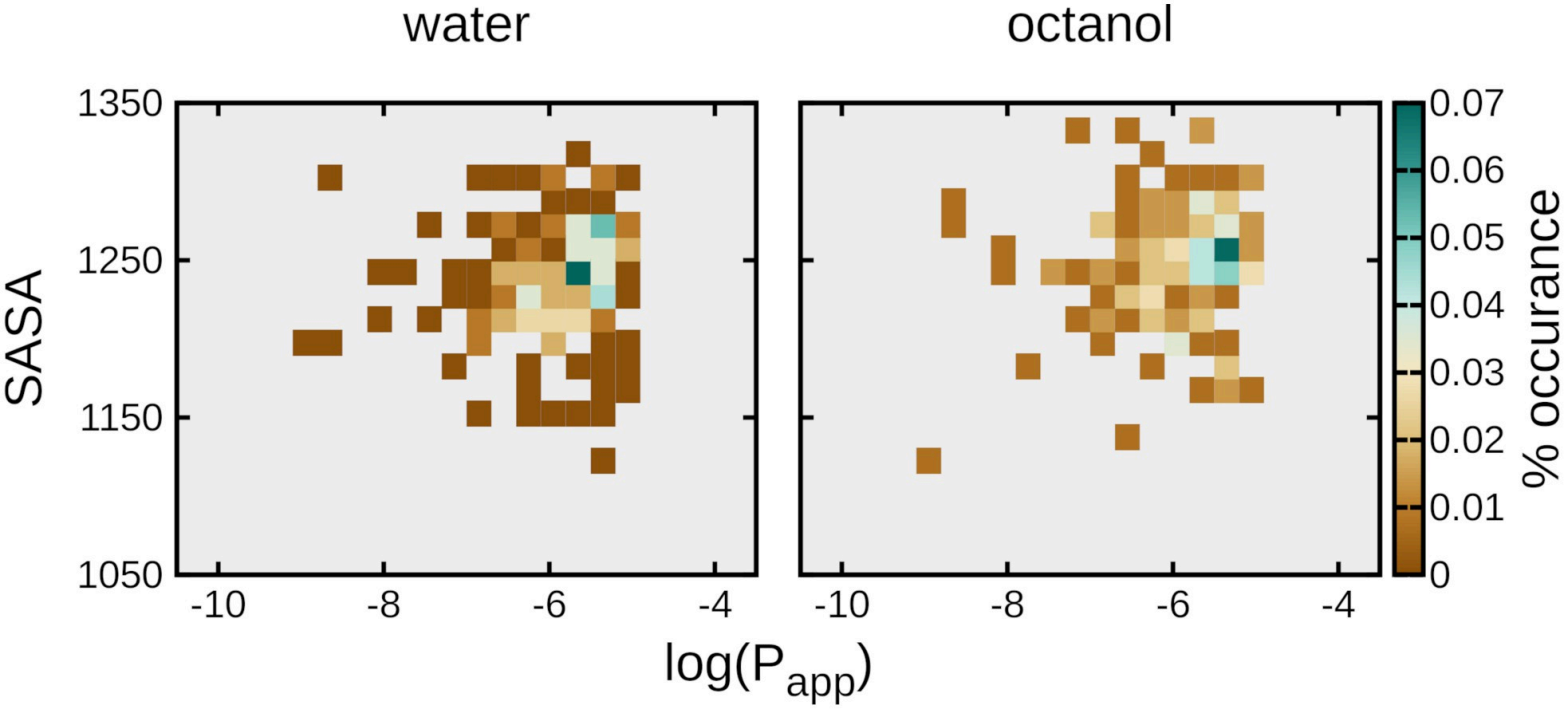
Changes in solvent-accessible surface area as a function of solvent is correlated with changes in log(P_app_). Average solvent-accessible surface area (SASA) over the entire trajectory for each respective peptide was calculated and plotted against the corresponding log(P_app_) from experimental studies.

Past studies have indicated that a potential correlation exists between solvent-accessible surface area (SASA) and permeability as a function of solvent [29]. The overall range of the distribution of SASA is roughly equivalent for both water and octanol, but there are differences in the nature of the distributions for each respective solvent (Fig 5). CPs in octanol have a SASA with a left-hand skew, leading to a more pronounced maximum at around 1250 A^2^ (S5 Fig). In contrast, CPs in water have a roughly normal distribution. The relationship of distributions between two solvents is more pronounced for less lipophilic values (log(P) > -6), which is relevant from a pharmacokinetics perspective. This less lipophilic segment of the population has a much more pronounced maxima in octanol, indicating that there is a decrease in conformational flexibility of the peptides which then leads to a narrowed range of sampled SASAs. All of these descriptions are rather general, reflecting the difficult nature of identifying correlative data within our simulations. This is not an uncommon issue in computational studies of CPs; large-scale computational studies of CPs have often needed to categorize the peptides into smaller cohorts by specific conformational characteristics (e.g., cage-like, beta-turn, or collapsed beta-turn) to identify correlative behavior within specific types of solvent [26, 29].

Even though we can observe quantifiable differences in behavior of SASA between solvents, we do not have enough resolution to achieve a true characterization of different conformational behavior as a function of solvent. Principal component analysis (PCA) is an approach that can qualitatively distinguish between conformational differences of lariat peptides as a function of solvent. In the case of CPs, PCA is most effective when applied to the conformational space of backbone dihedral angles rather than Cartesian space, as dihedral-based PCA does not require removal of translation and rotation from the system [45, 46]. We carried out PCA analysis on each of the 160 solvent pairs for both Cartesian coordinates of backbone heavy atoms and backbone dihedrals, plotting them as a function of the first versus second principal components (S6 Fig). Upon visual inspection, we identified 10% of the peptides with distinct PC1/PC2 profiles as a function of solvent. Of these 16 peptides, a subset of six of them had experimental data for which we could generate a comparison between log(P) and log(P_app_) (S7 Fig). They are generally positively correlated (R^2^ = 0.40), indicating that PCA analysis could be a viable tool for identification of peptides with quantifiable conformational differences in aqueous and membrane-like solvents. This result is in general agreement with the workflow that has been established by Leidl and coworkers, where they used accelerated MD to efficiently sample conformational space of a smaller library of CPs to characterize the difference in behavior in water, chloroform, or DMSO [47, 48]. Two areas of improvement that we will be pursuing in future work will be to 1) validate on a much larger data set and 2) identify machine learning approaches that can be used to automate identification of peptides that show conformational changes between solvents.

## Discussion

Physics-based models for prediction of membrane permeability have been shown to be superior to QSAR-based models [25]. With the improved speed of MD simulations, the work presented here shows that we are able to capture the dynamic conformational behaviors of cyclic peptides in both water and octanol, providing a richer data set to identify drivers of permeability. Distinct conformational behavior is particularly evident in the increase of intramolecular hydrogen bonding in octanol (Fig 3), which indicates shielding of polar groups along the cyclic peptide backbone. This behavior would also be present in the hydrophobic environment of cellular membranes, leading to improved permeability via 1) a decrease in unfavorable electrostatic interactions and 2) an increase in molecular compactness. Existing MD methods to predict membrane permeability are computationally expensive [43, 49]; in contrast, our solvent-only simulations converge rapidly, allowing for high-throughput screening on a relatively modest compute environment. Admittedly, our approach leaves room for improvement with respect to correlation with experimental data, but it shows promise as a virtual high-throughput technique. Future work will address prediction quality by optimization of the following areas: 1) utilization of enhanced sampling techniques (GaMD or others) to ensure comprehensive sampling of phase space; 2) implementing machine learning (e.g. regression models) to predict permeability from the MD simulations; and 3) applying cheminformatics filters to efficiently select viable CP candidates [39, 50, 51].

Regarding the data presented here, the peptide modification with the largest impact on membrane permeability is the degree of methylation. As the degree of N-methylation increases, the CP backbone skews towards larger populations of conformations favoring a *cis* orientation. This is consistent with what was observed experimentally [52] and also what has been shown on other series of CPs [26]. Higher proportions of *cis* conformation within CPs are often desirable, leading to a propensity to mimic β-sheet conformations that are known to target G protein-coupled receptors [38]. The driver for this conformational shift most likely results from a reduction in hydrogen-bonding partners, allowing the N-methyl groups to rotate toward solvent. Overall exposure is more likely to induce a constrained conformation, we therozie this is a result of the reduction of hydrogen bonds allowing the methyl groups to rotate toward the solvent. Interestingly, peptides in organic solvents show large populations of *cis*-amide conformation at low conformations until the total number of methylated sites are *N* > 2.

One aspect of our study that merits further investigation is that the inclusion of D-amino acids in lariat peptides contradicts the current *status quo* of the cyclic peptide field with respect to total solubility (i.e., we see no effect as a function of the number of D-amino acids). This is not to say that the presence of D-amino acids fails to influence membrane diffusion, [53], but that their effect may be subtle and our solvent-based approach may fail to include a sufficient level of detail to capture these subtleties. Modeling of CPs containing D-amino acids warrants inclusion of more realistic membrane systems. Solvent-based simulations are generally most useful for quickly eliminating non-viable structures for larger data sets (hundreds to thousands of CPs). Higher-level simulations (e.g., explicitly including lipid bilayers, applying enhanced sampling approaches like umbrella sampling) could then be applied as a post-screening step to obtain a more detailed characterization of how individual CPs passively diffuse across the membrane.

## Conclusion

We have presented here a relatively cost-effective physics-based approach to predicting permeability of lariat peptides. While it produced promising results, there are clearly areas for improvement to our model, which forms the basis of ongoing research in our lab. However, the framework exists to apply this approach to cyclic peptides in general and subsequently coupling these results to ML approaches to predict permeability behavior of a significantly expanded set of peptides. Thus, the techniques presented here will prove beneficial for high-throughput virtual screening of CP drug candidates in the future.

## Supporting information

S1 Fig Calculated log(P) values from MD simulations are in poor agreement with experimental data. Plot of the log of apparent partitioning coefficients (P_app_) obtained from [22] against partitioning coefficients calculated from simulations conducted in this study (log(P_calc_)).
(TIF)

S2 Fig Distribution of peptide library by N-methylation is roughly gaussian and indicates how oversampling of *N* = 0,4 groups is beneficial to understanding overall trends. Bar plot of the number of cyclic peptides that were tested according to N-methylation for the experimental approach in [22] (*dark gray*) and the simulation-based approach (*light gray*). Data recovery percentage is based on the expected number of cyclic peptides that could theoretically be produced during library generation versus the actual number of cyclic peptides that were synthesized [22].
(TIF)

S3 Fig Heterochirality in general has no effect on calculated permeability of cyclic peptides. Box plot of partition coefficients based on MD simulations (log(P_calc_)) as a measure of the degree of heterochirality (i.e., number of D-amino acid residues). Mid-points in each box is the mean for each particular data set. Boxes represent the two intermediate quartiles of each data set.
(TIF)

S4 Fig Cyclic peptides tested by heterochirality is skewed towards peptides with two to five D-amino acids. Histogram of cyclic peptides in this study based on degree of heterochirality.
(TIF)

S5 Fig Projection and 3D histogram of solvent-accessible surface area (SASA) as a function of solvent. **A** 2D projection of SASA of lariat peptides in water (*blue*) or octanol (*orange*). **B)** Three dimensional relationship of log(P_app_) vs. SASA vs. probability of lariat peptides in water (*left*) or octanol (*right*).
(TIF)

S6 Fig PCA can be used to identify peptides with distinct conformational behavior between aqueous and organic solvent. Principal component analysis (PCA) was applied to the heavy atoms in cyclic peptide backbones, plotting the first (PC1) versus second (PC2) principal component in the PCA series. *Blue*: water; *yellow*: octanol.
(TIF)

S7 Fig Filtering of data via PCA leads to reliable correlation between experimental and predicted peptide permeability. Plot of the log of apparent partitioning coefficients (P_app_) obtained from [22] against partitioning coefficients calculated from simulations conducted in this study (log(P_calc_)) using only cyclic peptides that displayed distinct behavior from PCA (S6 Fig).
(TIF)

S1 Table Lariat peptides used for visual correlation of principal component analysis as a function of solvent. (TIF)
