# Supplementary figures and images for: Gaussian accelerated molecular dynamics simulations facilitate prediction of the permeability of cyclic peptides
Source: PLoS One. 2024 Apr 23;19(4):e0300688. doi: 10.1371/journal.pone.0300688 (PMC11037548; doi:10.1371/journal.pone.0300688)

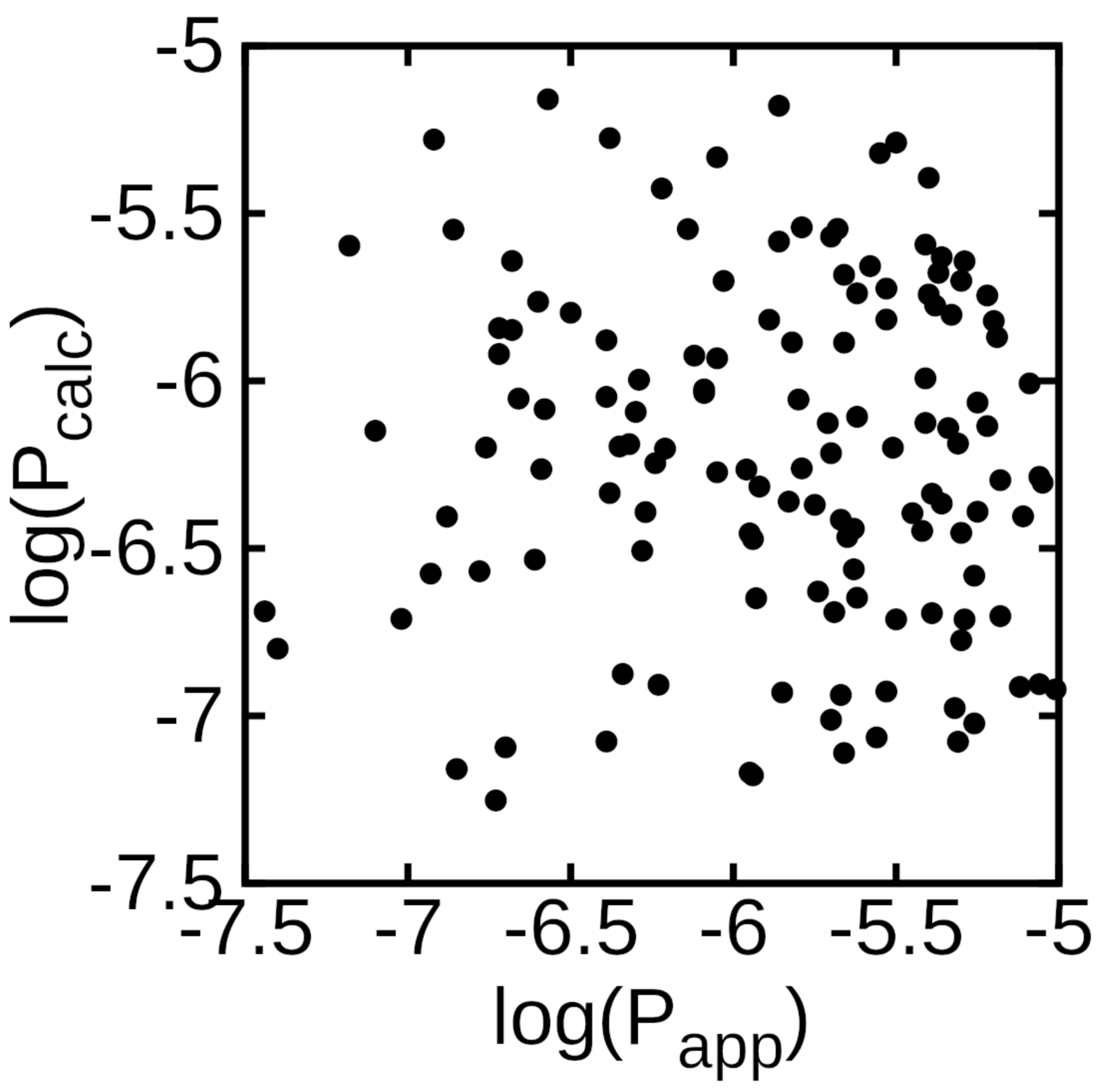

Supplement: S1 Fig — Plot of the log of apparent partitioning coefficients (Papp) obtained from [22] against partitioning coefficients calculated from simulations conducted in this study (log(Pcalc)). (TIF) [file pone.0300688.s001.tif]

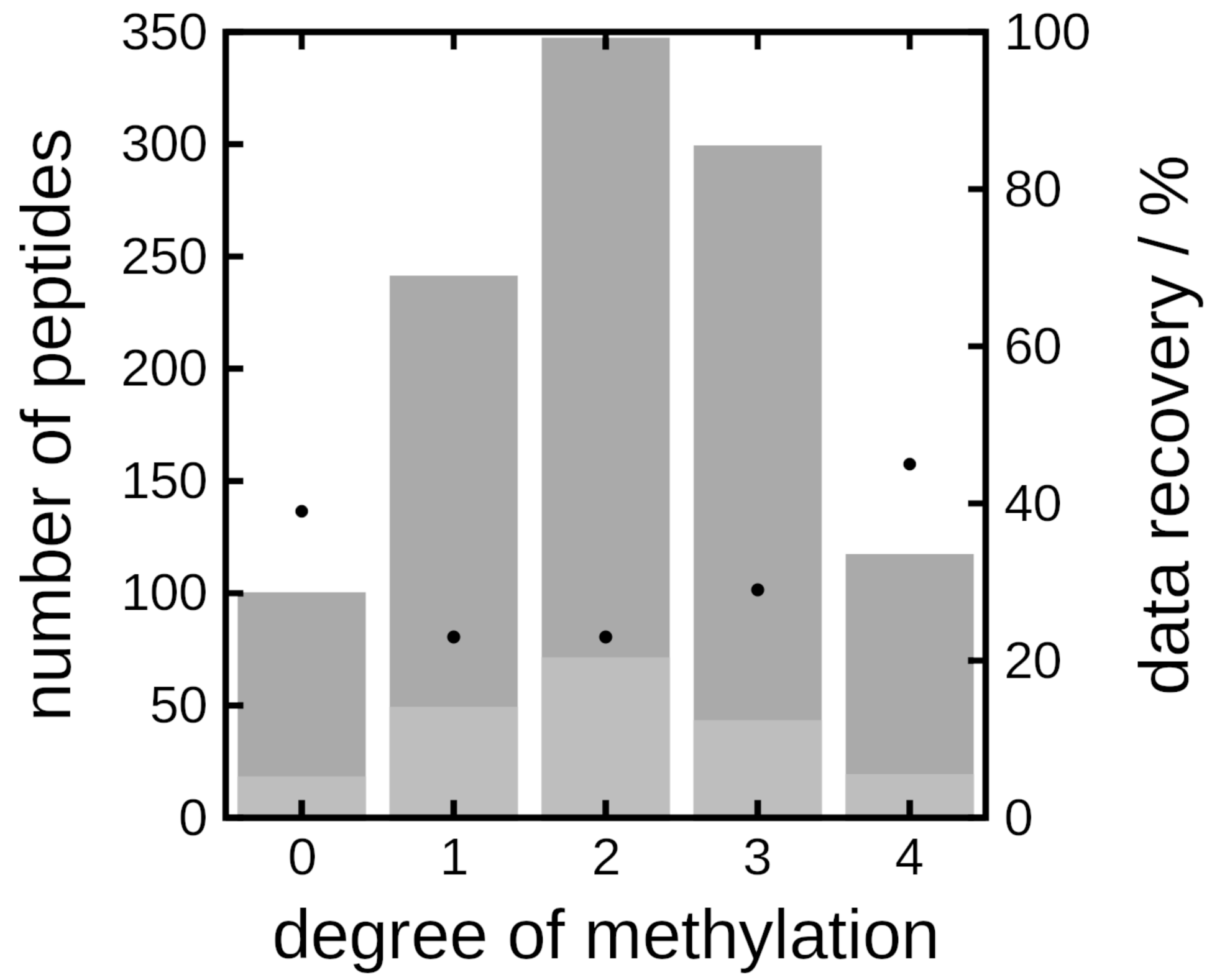

Supplement: S2 Fig — Bar plot of the number of cyclic peptides that were tested according to N-methylation for the experimental approach in [22] (dark gray) and the simulation-based approach (light gray). Data recovery percentage is based on the expected number of cyclic peptides that could theoretically be produced during library generation versus the actual number of cyclic peptides that were synthesized [22]. (TIF) [file pone.0300688.s002.tif]

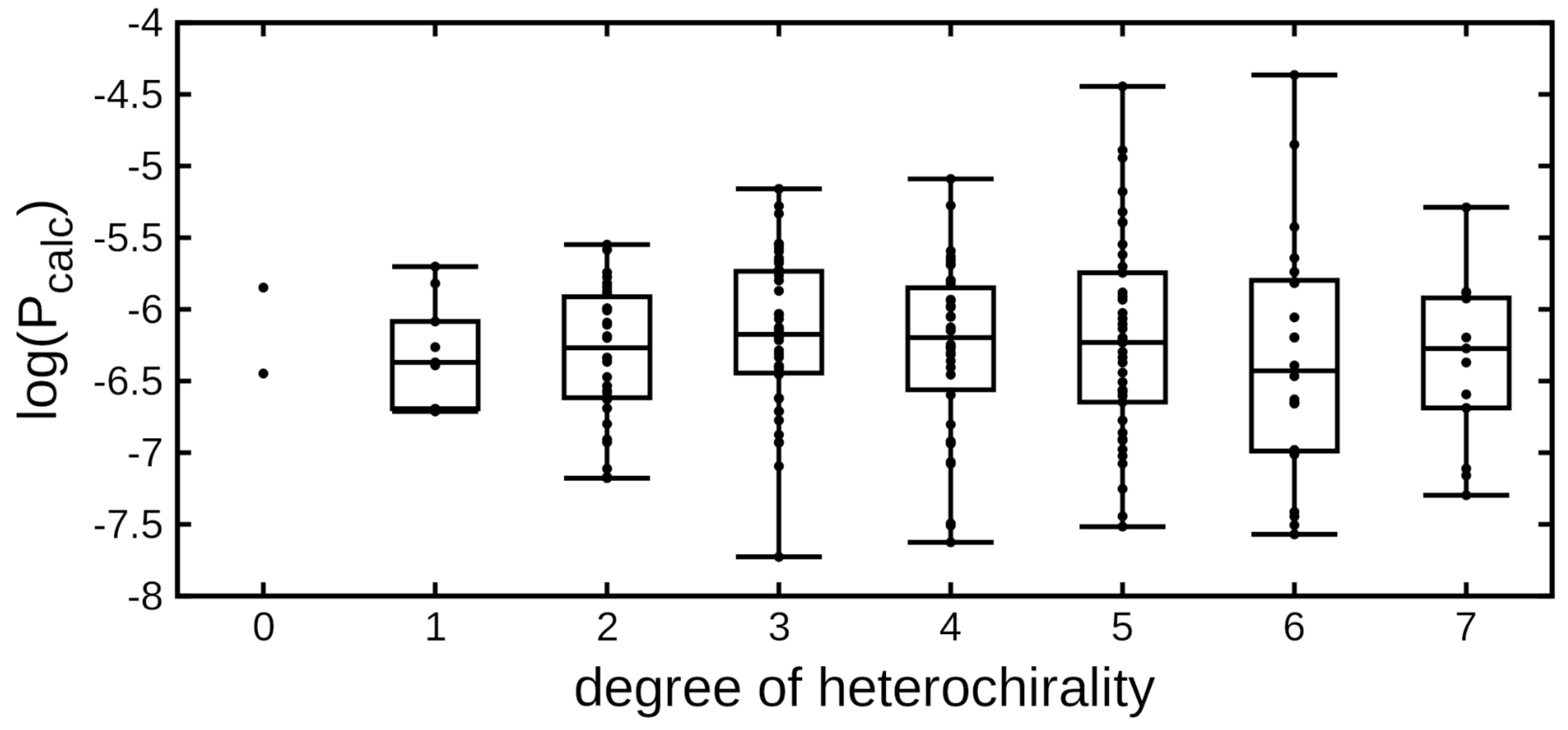

Supplement: S3 Fig — Box plot of partition coefficients based on MD simulations (log(Pcalc)) as a measure of the degree of heterochirality (i.e., number of D-amino acid residues). Mid-points in each box is the mean for each particular data set. Boxes represent the two intermediate quartiles of each data set. (TIF) [file pone.0300688.s003.tif]

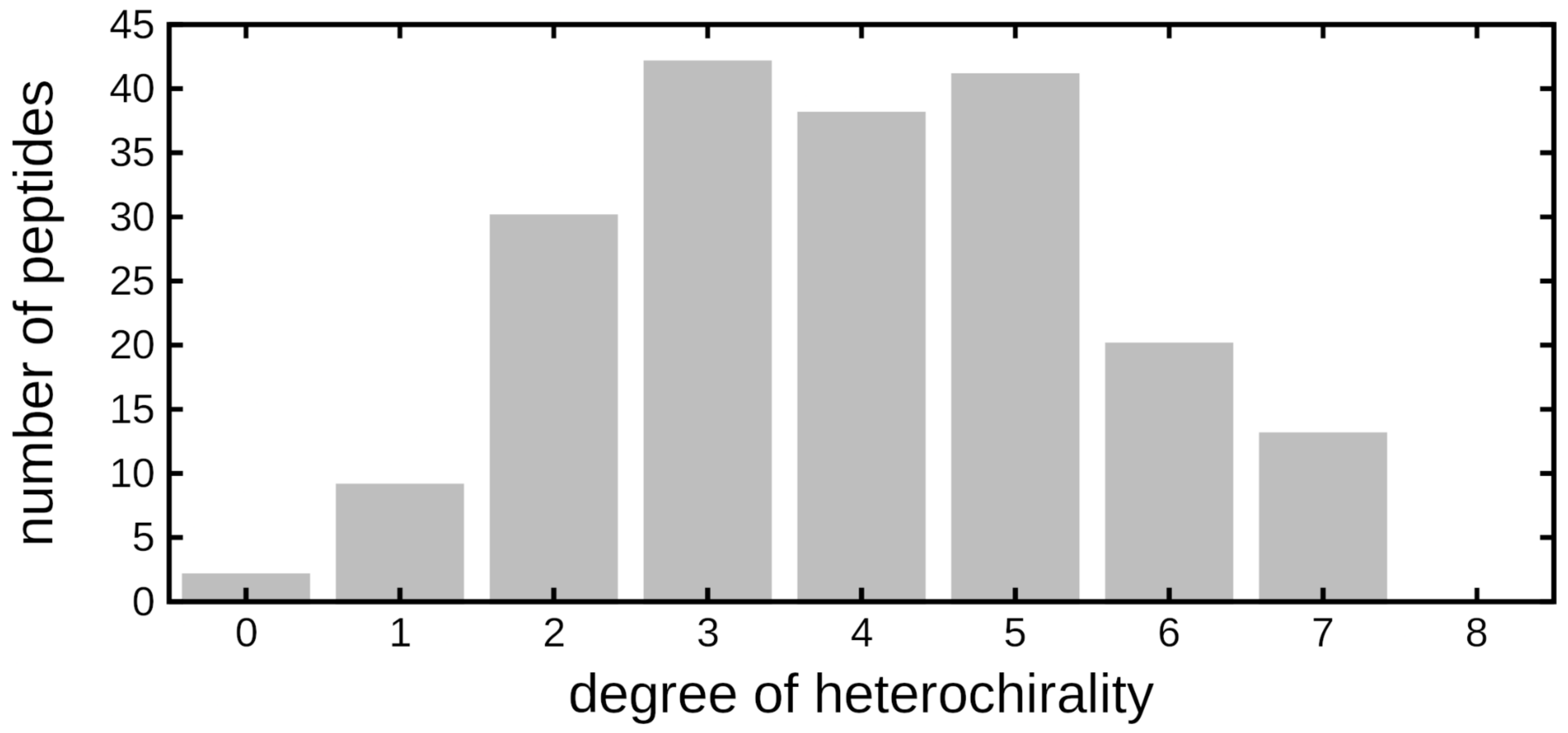

Supplement: S4 Fig — Histogram of cyclic peptides in this study based on degree of heterochirality. (TIF) [file pone.0300688.s004.tif]

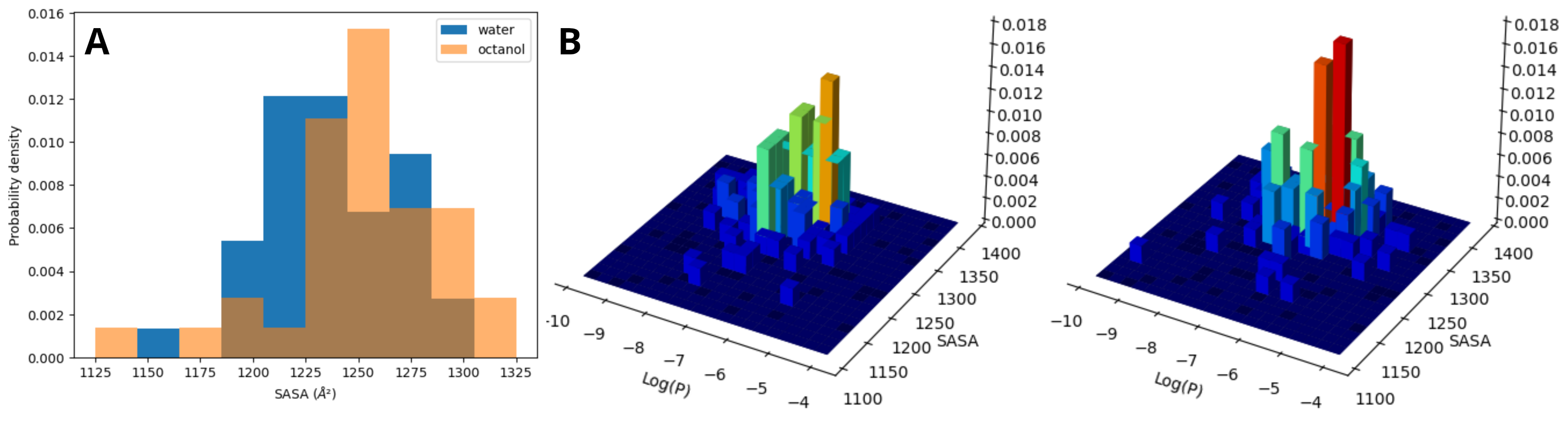

Supplement: S5 Fig — A 2D projection of SASA of lariat peptides in water (blue) or octanol (orange). B) Three dimensional relationship of log(Papp) vs. SASA vs. probability of lariat peptides in water (left) or octanol (right). (TIF) [file pone.0300688.s005.tif]

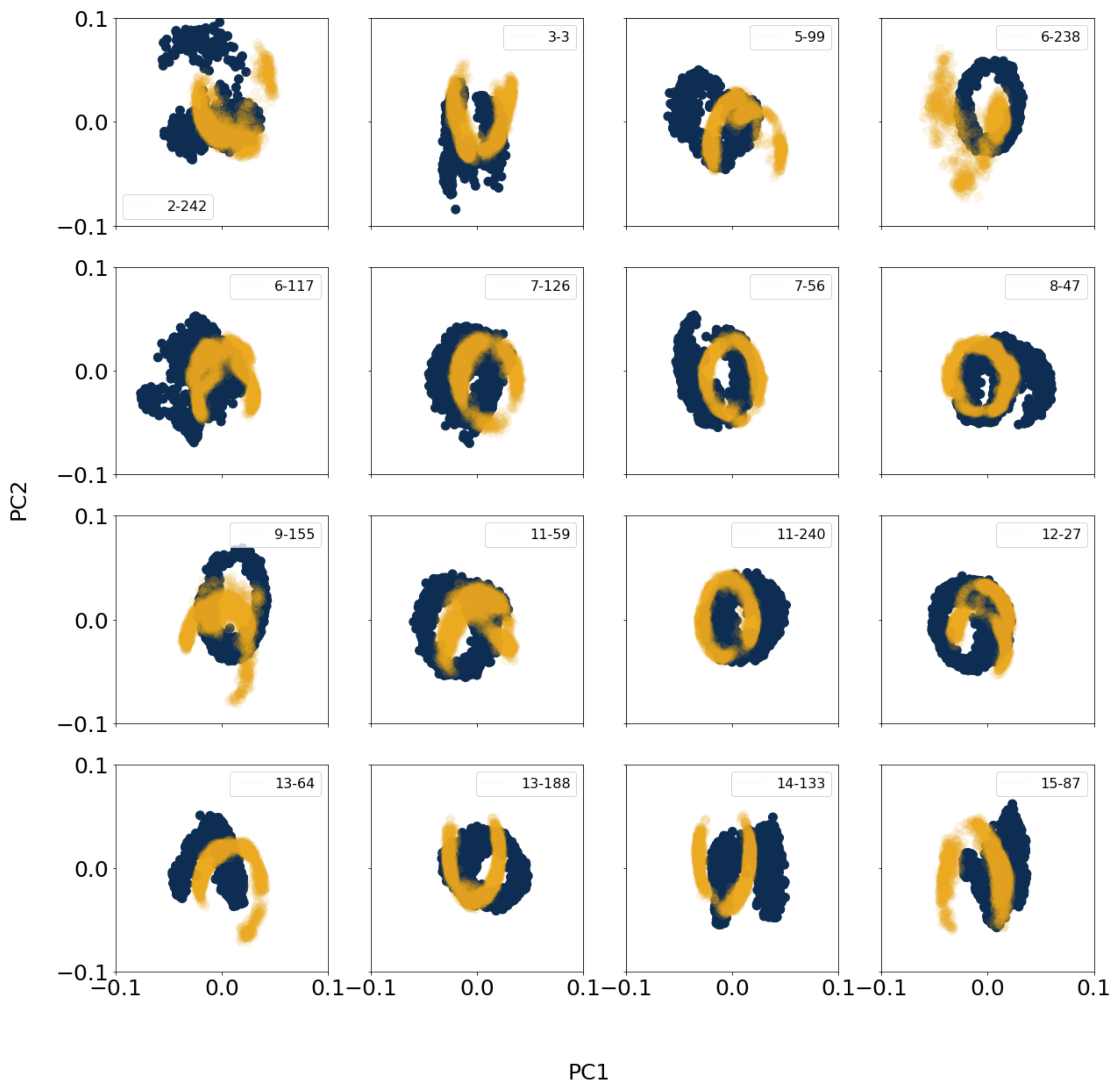

Supplement: S6 Fig — Principal component analysis (PCA) was applied to the heavy atoms in cyclic peptide backbones, plotting the first (PC1) versus second (PC2) principal component in the PCA series. Blue: water; yellow: octanol. (TIF) [file pone.0300688.s006.tif]

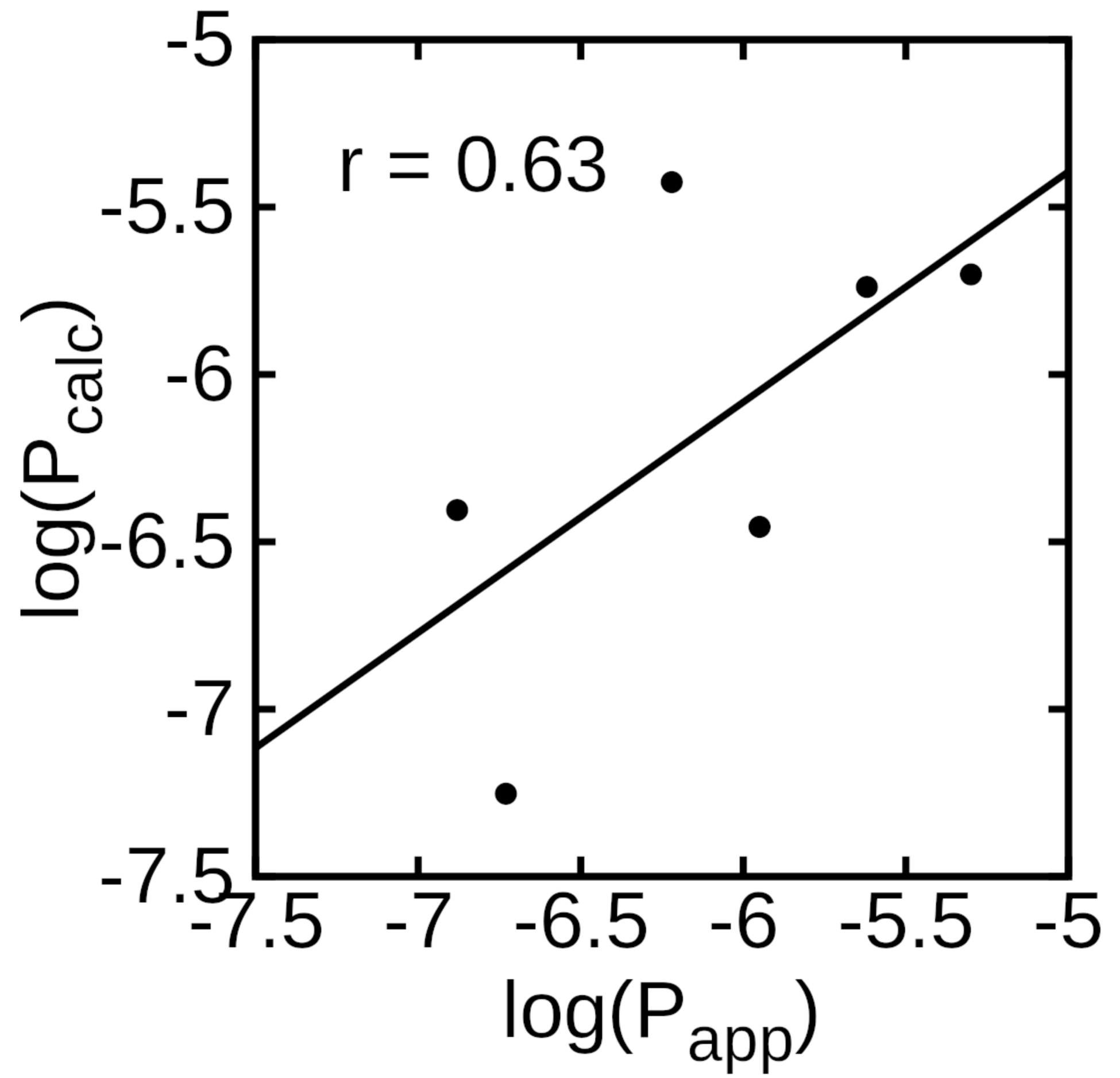

Supplement: S7 Fig — Plot of the log of apparent partitioning coefficients (Papp) obtained from [22] against partitioning coefficients calculated from simulations conducted in this study (log(Pcalc)) using only cyclic peptides that displayed distinct behavior from PCA (S6 Fig). (TIF) [file pone.0300688.s007.tif]

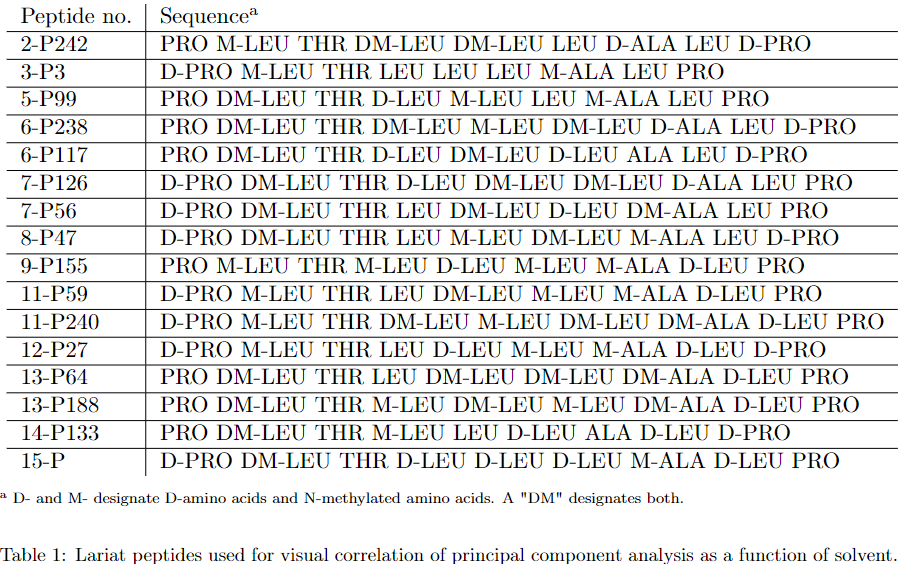

Supplement: S1 Table — (TIF) [file pone.0300688.s008.tif]
